# Opitz trigonocephaly syndrome presenting with sudden unexplained death in the operating room: a case report

**DOI:** 10.1186/1752-1947-5-222

**Published:** 2011-06-21

**Authors:** Laura Travan, Vanna Pecile, Mariacristina Fertz, Antonella Fabretto, Pierpaolo Brovedani, Sergio Demarini, John M Opitz

**Affiliations:** 1 Neonatal Intensive Care Unit, Institute for Maternal and Child Health Burlo Garofolo, Via dell'Istria 65/1, 34100, Trieste, Italy; 2 Department of Genetics, Institute for Maternal and Child Health Burlo Garofolo, Via dell'Istria 65/1, 34100, Trieste, Italy; 3 Departments of Pediatrics, Human Genetics, Obstetrics, and Gynecology, University of Utah, Salt Lake City, UT, USA

## Abstract

**Introduction:**

Opitz trigonocephaly C syndrome (OTCS) is a rare malformation syndrome with the following features: synostosis of metopic suture, craniofacial abnormalities, severe mental retardation and a multitude of pathological findings affecting almost every organ system. OTCS is associated with a high mortality rate.

**Case presentation:**

We describe the case of a Caucasian male baby who died at five months of age during surgical correction of the craniofacial anomaly.

**Conclusion:**

As previously reported, OTCS may have an increased mortality rate during craniofacial surgery. Careful evaluation of surgery risk-benefit ratio is warranted in such patients.

## Introduction

Opitz trigonocephaly C syndrome (OTCS) is a rare and heterogeneous genetic disorder characterized by synostosis of metopic suture, dysmorphic facial features, variable mental retardation and other congenital somatic and cerebral anomalies. Morbidity and mortality are very high. Fewer than 60 cases have been reported in the literature, mostly as single case reports or small series. We describe a white male baby who died at five months of age during surgery performed to correct the craniofacial anomaly.

## Case Presentation

Our patient was a Caucasian baby, born to nonconsanguineous parents at 39 weeks of gestational age. This was the first pregnancy of a 30-year-old mother with a bicornuate uterus. Pregnancy was complicated by early intrauterine growth retardation; antenatal ultrasound assessment was otherwise reported as normal.

Labor and delivery were spontaneous. The Apgar score was 9 and 10, respectively at one and five minutes. Birth weight was 2470 g (< 3rd percentile, small for gestational age), length was 46.7 cm (3^rd ^to 10th percentile), head circumference 33.1 cm (10th percentile).

**Figure 1:**
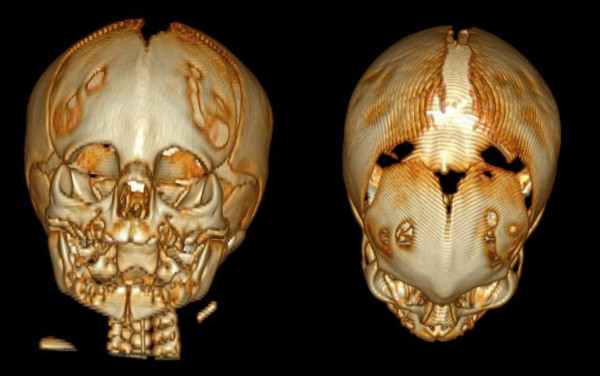
**Three-dimensional computerized tomography**. See the fusion of metopic suture.

At birth there was a marked trigonocephaly and other dysmorphic craniofacial features: micrognathia, upslanting eyelids, hypotelorism, depressed nasal bridge, low set ears. Cardiac and renal ultrasounds were normal. Computed tomography confirmed the early closure of metopic suture (Figure 1). Initially the baby was fed by nasogastric tube. At discharge after one week, he was fed completely by bottle.

**Figure 2:**
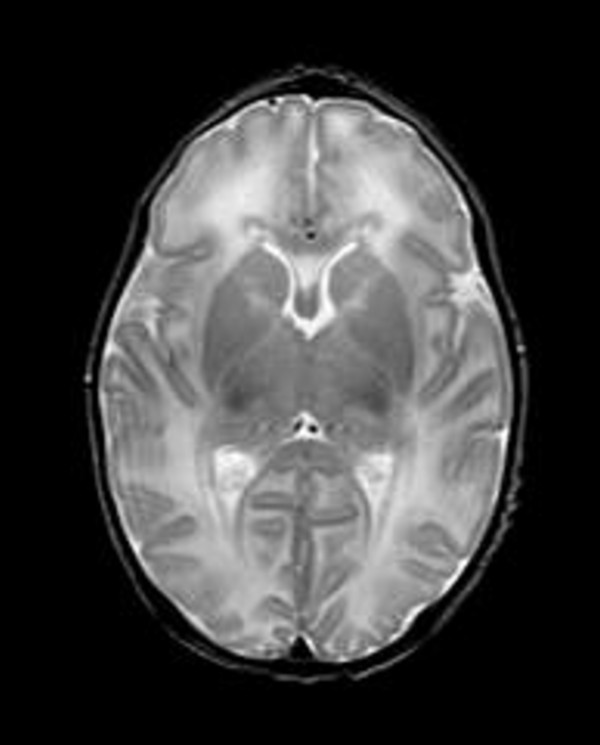
**Cerebral MRI (whitened T2 sequences), showing diffuse white matter periventricular hyperintensity (hypointensity in T1 sequences)**.

At 40 wks post-conceptional age brain MRI showed a small area of hyper-intensity under the posterior horn of the left ventricle (interpreted as calcification of a periventricular hemorrhage) and a diffused alteration of white periventricular matter (Figure 2).

An auditory brain stem response (ABR) test performed at 44 weeks revealed an absent pattern on the left ear.

Clinical evaluation during the first four months of life did not show an evident psychomotor delay; however fidgety activity seemed absent.

Chromosome analysis showed a normal 46 XY karyotype. We also performed single-nucleotide polymorphism (SNP) array without any significant finding.

The baby died unexpectedly at five months of age during surgery performed to correct the craniofacial anomaly. Autopsy did not add anything to the clinical picture: specifically, there were no additional anomalies except for a double left renal artery. Some micro calcifications were found around brain vessels.

## Discussion

OTCS, first described in 1969 by Opitz [1] is characterized by trigonocephaly, mental retardation, short neck, typical facial appearance, joint and limb anomalies, up-slanting palpebral fissures, epicanthal folds, a broad depressed nasal bridge, small nose, abnormally low-set ears, and central nervous system and visceral anomalies, such as renal and heart anomalies.

OTCS is a heterogeneous genetic disorder which occurs sporadically, although familial cases have also been reported [2,3].

A very high mortality rate has been described: almost 50% of patients with OTCS die within the first year of life [3]. Some patients, however, may have a good quality of life: Patient 2 of Lalatta [4] has normal IQ. She underwent multiple craniosynostectomies but she did well at the University and was also able to play the piano.

Our patient had many of the clinical and anatomic findings typical of OTCS: the dysmorphic face, white matter alteration, as described by Lalatta [4] and by Azimi [5], cerebral hemorrhage [3] and hearing loss as reported by Nacarkucuk et al. [6] and Zampino et al. [7].

We did not find any genetic abnormality either in the karyotype or in the region of CD96 gene, as recently described by Kaname [8].

To the best of our knowledge this is the second case after patient 1 reported by Opitz [3] who died after surgery for craniosynostosis repair. That patient, after the skull reconstruction, developed hematuria, cardiac arrhythmia and severe acidosis requiring cardiopulmonary resuscitation. Twenty minutes later, he developed a severe intra-vascular coagulation. After the autopsy, experts in genetics, immunology and rheumatology concluded that patient 1 of Optiz had a possible connective tissue abnormality and increased vascular fragility that started the catastrophic cascade that led to death.

Our patient died under the same circumstances as patient 1 described by Optiz. Autopsy did not find vascular malformation or connective tissue anomalies that could have explained death during surgery. However, as in Opitz's patient 1 the cause of death was an unexpected massive bleeding.

## Conclusion

OTCS is a complex and heterogeneous condition that is still under-recognized and under-diagnosed. The fact that two children died as a consequence of craniofacial surgery may have clinical implications: diagnosing OTCS in trigonocephalic patients before surgery, may allow a better evaluation of risks and benefits of craniosynostosis repair.

## Consent

Written informed consent was obtained from patient's next-of-kin for publication of this case report and accompanying images. A copy of the written consent is available for review by the Editor-in-Chief of this journal.

## Competing interests

The authors declare that they have no competing interests.

## Authors' contributions

LT made the patient diagnosis confirmed by JMO; they and SD were major contributors in writing the manuscript. VP and FA performed and interpreted the genetic analysis. MF and PB performed clinical evaluations. All authors read and approved the final manuscript.
